# Using Open-Source Large Language Models to Identify Access to Germline Genetic Testing in Veterans With Breast Cancer From Unstructured Text

**DOI:** 10.1200/CCI-24-00263

**Published:** 2025-07-22

**Authors:** Chunyang Li, Michael Stringer, Vikas Patil, Richard Mcshinsky, Deborah Morreall, Christina Yong, Kelli M. Rasmussen, Zachary Burningham, Suzanne Tamang, Carolyn S. Menendez, Akiko Chiba, Haley A. Moss, Sarah Colonna, Kerry Rowe, Daphne Friedman, Michael J. Kelley, Ahmad Halwani

**Affiliations:** ^1^ George E. Wahlen Veterans Affairs Medical Center, Salt Lake City, UT; ^2^ University of Utah School of Medicine, Salt Lake City, UT; ^3^ School of Medicine, Stanford University, Stanford, CA; ^4^ Department of Veterans Affairs, Menlo Park, CA; ^5^ Duke University School of Medicine, Durham, NC; ^6^ Durham Veterans Affairs Health Care System, Durham, NC; ^7^ Department of Veterans Affairs (VA), National Oncology Program, Washington, DC; ^8^ Huntsman Cancer Institute, Salt Lake City, UT

## Abstract

**PURPOSE:**

The ability of large language models (LLMs) to identify access to germline genetic testing from unstructured text remains unknown. The Department of Veterans Affairs (VA) assessed access in Veterans with breast cancer by implementing and evaluating the performance of open-source, locally deployable LLMs (Llama 3 70B, Llama 3 8B, and Llama 2 70B) in identifying access from clinical/consult notes.

**METHODS:**

We identified a cohort of 1,201 Veterans diagnosed with breast cancer between January 1, 2021, and December 31, 2022, who received cancer care within the nationwide VA system and had clinical and/or consult notes available. Notes from a subset of 200 randomly selected patients, reviewed by subject-matter experts to identify access to testing, were split into development and testing sets, and various hyperparameters and prompting approaches were applied. We evaluated LLM performance using accuracy, precision, recall, and F1, with expert consensus on the labeled subset serving as ground truth. We compared LLM-identified access distribution in the entire cohort with expert-identified access in the labeled subset using the chi-squared test.

**RESULTS:**

Llama 3 70B achieved an F1 score of 0.912 (95% CI, 0.853 to 0.971), besting Llama 3 8B (F1: 0.811; 95% CI, 0.720 to 0.901) and significantly outperforming Llama 2 70B (F1: 0.644; 95% CI, 0.514 to 0.773; the test set target variable prevalence was 0.72.) We observed no significant difference between the performance of Llama 3 70B and that of the average individual expert reviewer, nor between LLM-identified access distribution across the entire cohort and expert-identified distribution in the labeled subset.

**CONCLUSION:**

An open-source, locally deployable LLM effectively and efficiently identified germline genetic testing access from clinical notes. LLMs may enhance care quality and efficiency, while safeguarding sensitive data.

## INTRODUCTION

Breast cancer is the most common cancer in women and the second leading cause of cancer-related mortality in women in the United States.^1,2^ Five to 10% of women diagnosed with breast cancer in the United States will have inherited a pathogenic germline variant (PGV).^3^ High-penetrance genes such as *BRCA1* and *BRCA2* (*BRCA1/2*), *TP53*, *PTEN*, *CDH1*, *STK11*, and *PALB2* increase the risk of breast cancer more than four times, whereas moderate-penetrance genes such as *ATM*, *NF1*, and *CHEK2* increase risk two to three times.^4^ Identifying germline mutations significantly affects cancer prevention, early detection, and treatment for patients and their relatives with an identified PGV. In 2019, the American Society of Breast Surgeons recommended universal germline genetic testing for all patients with breast cancer.^5^ However, the majority of eligible patients never receive testing, because of barriers such as a lack of provider or patient awareness, concerns over cost and discrimination, and limited access to genetic counseling.^4^

**CONTEXT**

**Key Objective**
Can a large language model (LLM) identify access to germline genetic testing from clinical notes?
**Knowledge Generated**
This work investigated the feasibility of using open-source LLMs to extract information from unstructured electronic health care record data to identify access to germline genetic testing in Veterans with breast cancer. The Llama 3 70B LLM achieved an F1 score of 0.912 (95% CI, 0.853 to 0.971), comparable with the collective accuracy of subject-matter expert reviewers.
**Relevance *(F.P.-Y. Lin)***
Locally deployable open-source LLMs can accurately identify access to germline genetic testing from clinical notes, offering healthcare systems a privacy-preserving solution to efficiently monitor guideline adherence and improve identification of patients who may benefit from hereditary cancer risk assessment.*
*Relevance section written by *JCO Clinical Cancer Informatics* Associate Editor Frank P.-Y. Lin, PhD, FRACP, MBChB, FAIDH.

The population of women serving in the military has grown rapidly, with one in five Veterans being women.^6^ They represent the fastest-growing population using Department of Veterans Affairs (VA) health care,^7^ and the number of women with breast cancer receiving VA care has increased steadily. Breast cancer is the most common cancer among women Veterans age 20-59 years. From 1995 to 2012, the number of women diagnosed with breast cancer at the VA more than tripled.^8^ The VA has implemented several programs to ensure access to germline genetic testing for Veterans, including the Clinical Cancer Genetics Service, the National Precision Oncology Program, and the Breast and Gynecologic Cancer System of Excellence.^9-11^

The Making Advances in Mammography and Medical Options for Veterans Act (Public Law 117-135, known as the MAMMO Act, signed on June 7, 2022) aims to enhance the quality of VA mammography services and improve health care for Veterans, focusing on breast cancer screening and early detection.^12^ MAMMO Act Section 104 mandates a study on the availability of breast cancer genetic testing among Veterans and calls for expanding access to such testing.

To address this mandate, the VA National Oncology Program (NOP) conducted a study identifying the prevalence of genetic testing within the VA. Findings will be detailed in a separate publication.^13^ Briefly, a team of health information technology experts and subject-matter experts (SMEs), including VA breast cancer providers and VA NOP stakeholders, collaborated to define study objectives, identify the relevant Veteran population, and outline clinical workflows. Using a health information technology approach informed by the sociotechnical model,^14^ the team defined data products and analytics and developed a user interface using lean principles to facilitate rapid review of clinical notes from 200 randomly selected VA patients with breast cancer.^15^ The team of SMEs performed a manual review to identify whether each patient had been offered germline genetic testing. While this approach addressed the congressional mandate, it was time-consuming, costly, and not scalable for near real-time, VA-wide characterization of germline genetic testing, hindering continuous quality improvement.

Natural language processing (NLP) algorithms and deep learning are widely used in medical information extraction.^16^ However, rule-based NLP approaches often suffer from limited accuracy and generalizability, while deep learning requires large, labeled data sets for effective training and evaluation. By contrast, large language models (LLMs) have gained popularity for their broad applicability across various tasks.^17^ LLMs, based on transformer neural networks and trained on extensive unstructured text data, can be used without the need for task-specific training.^18^ Prompt engineering enhances the activation and response capabilities of LLMs without relying on large, labeled data sets or encountering overfitting issues.^19^ Generative pretrained transformers (GPTs), such as GPT-4 and its user interface, ChatGPT, excel in extracting clinical information from unstructured text reports in areas of medicine such as radiology and pathology.^20^ Attempts to apply LLMs to unstructured clinical text to identify suicidality, detect adverse events in endoscopy, diagnose seizures, and extract fundamental sleep parameters and other clinically relevant features have achieved varying levels of success.^21-25^ However, LLMs typically require transferring data to third-party servers, which can expose sensitive health care information. Open-source LLMs such as Llama 2 and Llama 3 can be deployed locally, safeguarding sensitive information. Therefore, we used prompt engineering on Llama models to automate and streamline the identification of germline genetic testing at the VA.

## METHODS

This work was performed as part of the Veterans Health Administration's continuous quality improvement efforts, approved by the VA NOP office. As an operational project, it was deemed exempt from Institutional Review Board review. It follows the Standards for Quality Improvement Reporting Excellence (SQUIRE 2.0) guidelines.

### Data

**FIG 1.**
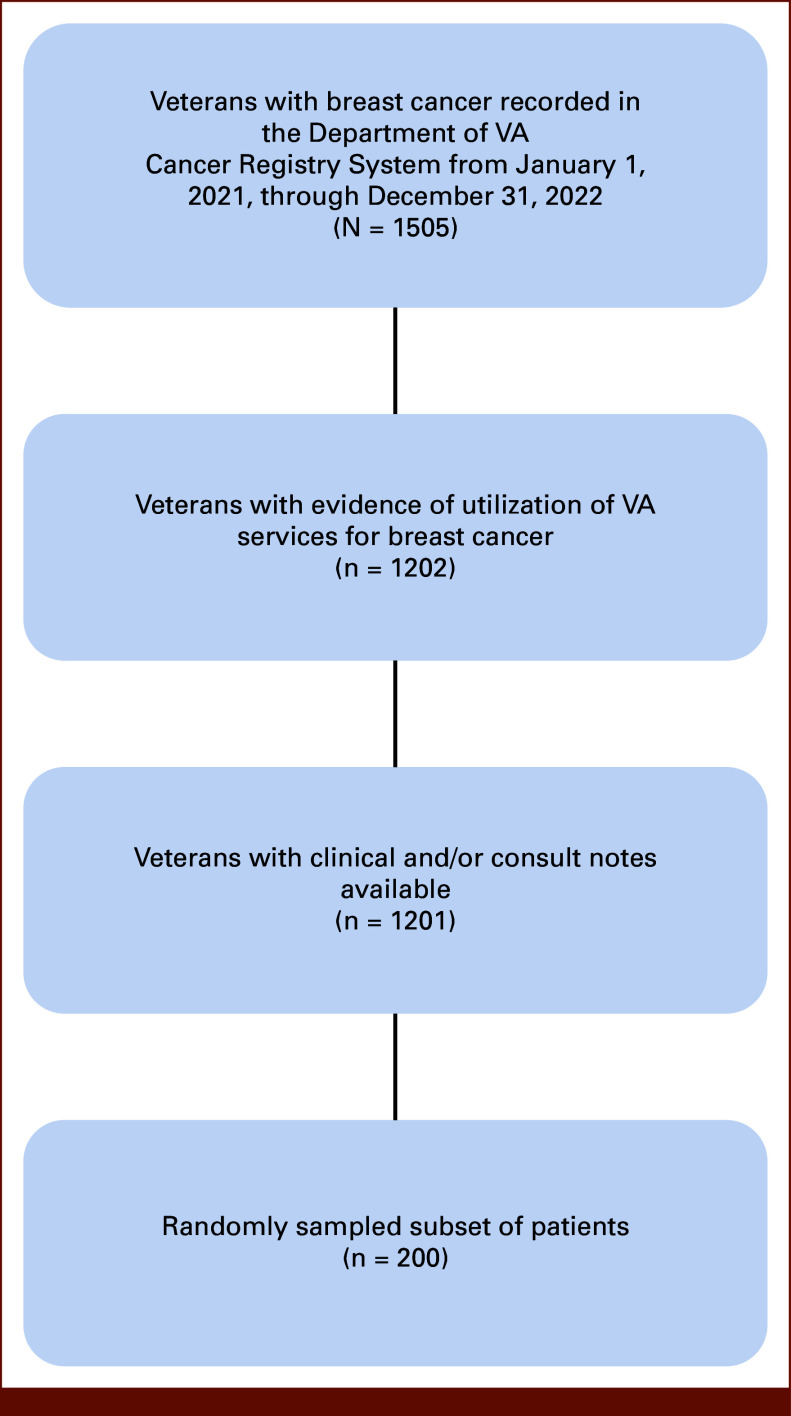
Patient cohort. VA, Veterans Affairs.

We identified Veterans with breast cancer diagnosed between January 1, 2021, and December 31, 2022, using data from the VA Cancer Registry System and VA Corporate Data Warehouse (CDW). Figure 1 illustrates inclusion criteria and the resulting set of patients. Two hundred patients randomly sampled from that group formed the cohort for this quality improvement project.

All data came from the VA CDW, which consolidates information from the VistA Computerized Patient Record System. It is highly accurate and represents all VA electronic health care record information. Our team of SMEs identified specialties relevant to breast cancer management and/or diagnosis. For each patient in the cohort, all documentation of any type for any relevant specialist visit was extracted. There were no inaccessible documents nor discrepancies.

The SMEs created a list of keywords that might indicate access to genetic testing; we also added keywords from reference texts about breast cancer genetic testing.^26,27^ Using Text Analytics for Health within the Microsoft Azure Government AI environment, we extracted text snippets containing 100 words before and after each keyword in clinical notes. To expedite chart review for the 200 patients, SMEs were shown the snippets, but could also view entire notes for context if desired. Each patient's documentation was independently reviewed by two reviewers, with any disagreements adjudicated by the entire SME team. This thorough process, designed to minimize the likelihood of introducing bias through processing or handling, yielded a labeled data set that we then used to develop and evaluate LLM performance in extracting information on access to genetic testing.

### Models

We evaluated the performance of three state-of-the-art, open-source LLMs: Llama 3 70B, Llama 3 8B, and Llama 2 70B. Llama 2 models with 7 billion (7B) and 70 billion (70B) parameters, respectively, were introduced in July 2023 and trained on 2 trillion tokens.^28^ Llama 3 models, trained on 15 trillion tokens, provide enhanced scalability and performance and support an 8k context length, doubling the capacity of Llama 2 models.^29^ The LLMs were run on Microsoft Azure Government Standard_ND96amsr_A100_v4 computers.

### Prompt Engineering

We tested zero-shot and few-shot prompting techniques using three styles—prefix (direct question), answering template, and cloze (fill in the blank)—on the development set.^30^ The best-performing prompt was zero-shot, using a template for the desired answer format. We reviewed error cases and refined prompts by adding specific instructions, which enhanced accuracy for the training set. Details about this process and its results are provided in the Appendix and Appendix Table A1.

### Evaluation

The labeled data set of 200 patients' notes was randomly divided into development and testing sets (50% each), both stratified by the four category labels and by whether there were any reviewer disagreements. This ensured similar distributions across both sets. We used the development set for refining prompts and adjusting LLM hyperparameters, whereas the final prompt and settings were applied to the test set for evaluation, to minimize the risk of overfitting and enhance generalizability.

For evaluation, the categories “No,” “Not sure,” and “Not applicable” were consolidated into a single “No” category. Categories were originally defined as follows:

1. Yes: The patient had germline genetic testing, received a recommendation of germline testing, or was referred through a consultation to VA Genomic Medicine Service/Clinical Cancer Genetics Service/community care for genetic counseling, AND the patient was seen by at least one relevant VA provider (eg, medical oncology, radiation oncology, surgery).
2. No: The patient was seen by at least one relevant VA provider and did not meet any of the criteria listed under “Yes”.
3. Not applicable: All breast cancer care was conducted in the community, as indicated by the patient not being seen by at least one relevant VA provider, regardless of the presence of the criteria listed under “Yes”.
4. Not sure: The patient was seen by a relevant VA provider and considered for germline genetic testing, but testing was not performed because the patient did not meet criteria. While this category could be grouped under “No,” it is separated to identify how many patients might have been tested under the current criteria.

We extracted LLM-generated responses using regular expressions, producing binary outputs. Accuracy, precision, recall, and F1 scores were then calculated to assess the performance of the prompts.

The final consensus labels, agreed upon by all reviewers, served as the ground truth. We also evaluated the performance of each individual reviewer's answers against the ground truth, using the same metrics applied to the LLMs.

## RESULTS

### Patients and Notes

Table 1 details the characteristics of the 200 randomly sampled patients whose charts were manually reviewed by SMEs. Among those 200 patients, 118 (59%) had access to germline genetic testing, whereas 82 (41%) did not. The median number of clinical notes per patient was 42 (IQR, 21-82). Patients had a median of 4 snippets containing relevant concepts (IQR, 1-10), with a median word count of 199 (IQR, 124-285) for all snippets. For patients with multiple snippets, these were consolidated into a single document for LLM processing.

**TABLE 1.** Patient Characteristics

Characteristic	Overall (N = 200)	Development Set (n = 100)	Testing Set (n = 100)	*P*
Age, years, mean (SD)	59.8 (10.9)	59.4 (11.4)	60.2 (10.4)	.632
Sex,^a^ No. (%)				
Female	185 (92.5)	89 (89.0)	96 (96.0)	.107
Male	15 (7.5)	11 (11.0)	4 (4.0)	
Race,^a^ No. (%)				
Hispanic	12 (6.0)	6 (6.0)	6 (6.0)	.609
Non-Hispanic Black	73 (36.5)	32 (32.0)	41 (41.0)	
Non-Hispanic White	95 (47.5)	51 (51.0)	44 (44.0)	
Other^b^	20 (10.0)	11 (11.0)	9 (9.0)	
Final decision with respect to access to testing, No. (%)				
No	61 (30.5)	31 (31.0)	30 (30.0)	.991
Not applicable	11 (5.5)	5 (5.0)	6 (6.0)	
Not sure	10 (5.0)	5 (5.0)	5 (5.0)	
Yes	118 (59.0)	59 (59.0)	59 (59.0)	
Initial disagreement during chart review by subject-matter experts, No. (%)				
No	171 (85.5)	86 (86.0)	85 (85.0)	1
Yes	29 (14.5)	14 (14.0)	15 (15.0)	
Geographic setting, No. (%)				
Rural	50 (25.0)	30 (30.0)	20 (20.0)	.142
Urban	150 (75.0)	70 (70.0)	80 (80.0)	

Abbreviation: SD, standard deviation.

a Self-reported.

b Includes American Indian or Alaska Native, Asian, Native Hawaiian or other Pacific Islander, unknown by patient, and patient declined to answer.

**FIG 2.**
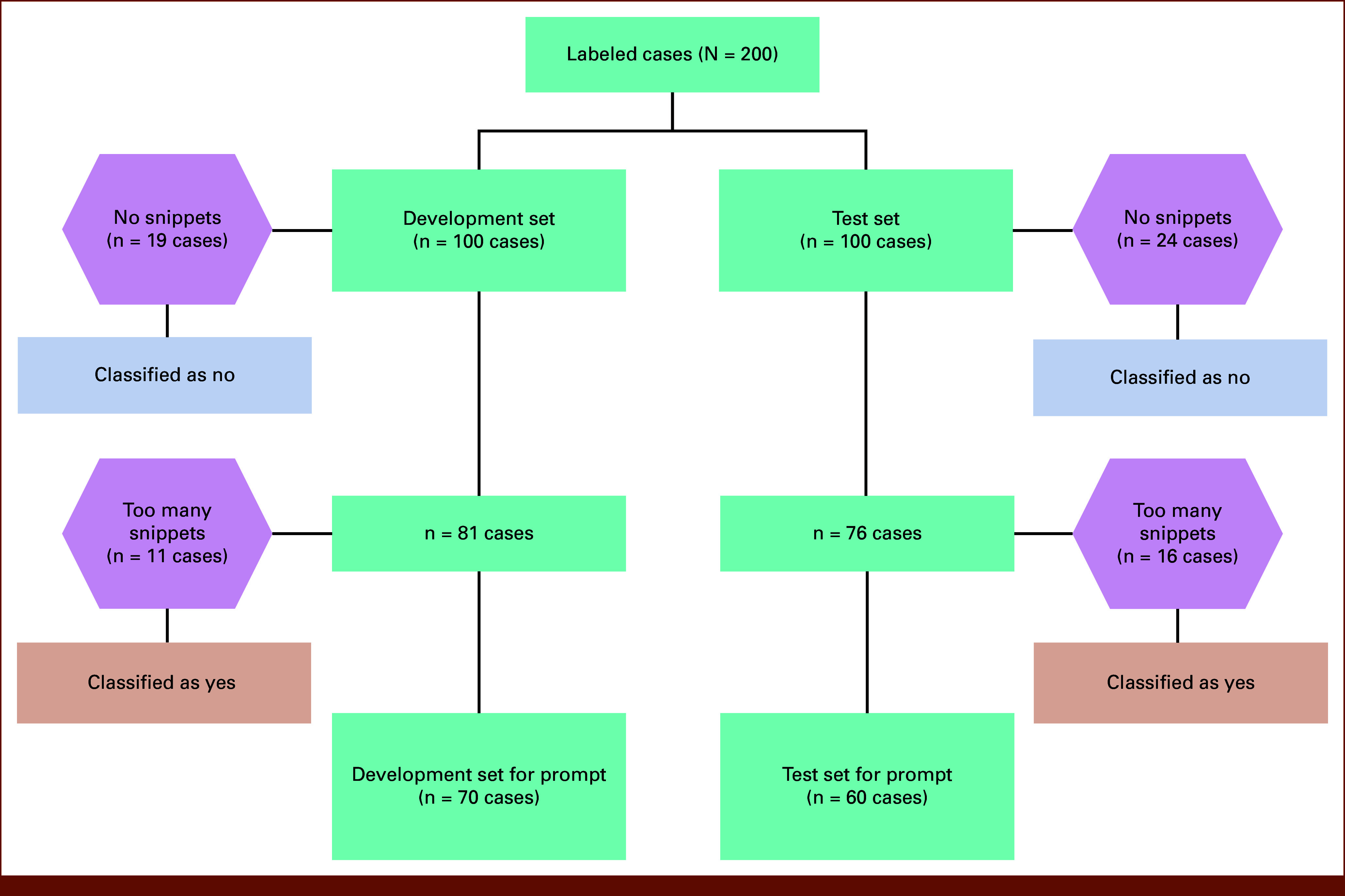
Case selection for large language model development and testing.

Forty-three patients with no snippets with relevant concepts in any notes were excluded from both the development and validation phases; none of these patients had access to germline genetic testing. Twenty-seven patients' notes either included at least 20 snippets or exceeded 4,000 words; all these patients had been offered testing. Thus, our final production pipeline included a pre-LLM step: patients without relevant concept mentions were automatically classified as not having been offered testing, and those with excessive concept mentions were automatically classified as having been offered testing (Fig 2). These patients were excluded from LLM processing and were not used for prompt development or testing.

Before tokenization, we cleaned consolidated documents by removing special characters and extraneous whitespaces. The median word count of the merged document was 800 (IQR, 310-1,554). Figure 2 illustrates the pipeline for case selection during development and testing.

### Performance

Table 2 presents performance metrics for Llama 3 70B, Llama 3 8B, and Llama 2 70B (used as a baseline) on the 60-case testing set (target variable prevalence 0.72). The temperature parameter for all models was set at 0.75, and the Top_p was set at 0.9. Mean performance metrics and CIs were calculated using 5,000 bootstrap iterations.

**TABLE 2.** Performance Comparison of Llama Models on the Testing Set

Metrics^a^	Llama 3 70B	Llama 3 8B	Llama 2 70B
Accuracy (95% CI)	0.875 (0.800 to 0.950)	0.754 (0.639 to 0.869)	0.615 (0.492 to 0.738)
F1 score (95% CI)	0.912 (0.853 to 0.971)	0.811 (0.720 to 0.901)	0.644 (0.514 to 0.773)
Precision (95% CI)	0.898 (0.816 to 0.979)	0.865 (0.757 to 0.972)	0.929 (0.857 to 1.000)
Recall (95% CI)	0.921 (0.842 to 1.000)	0.769 (0.643 to 0.895)	0.500 (0.354 to 0.646)
Execution time, seconds	251.95	21.42	977.02

a The prevalence of target variable in this 60-case testing set (for all Llama models) was 0.72.

Llama 3 70B performed best, achieving an F1 score of 0.912 (95% CI, 0.853 to 0.971), outperforming Llama 3 8B and significantly outperforming Llama 2 70B, which had an F1 score of 0.644 (95% CI, 0.514 to 0.773). The CIs for the performance metrics of Llama 3 70B and Llama 3 8B overlapped, likely because of the small testing set. Notably, Llama 2 70B not only underperformed compared with the similarly sized Llama 3 70B but also required considerably longer execution time (977 seconds for Llama 2 70B compared with 252 seconds for Llama 3 70B). Llama 3 8B, the smallest model, executed much faster with parallel distributed processing.

Based on these results, we selected Llama 3 70B for our task. Table 3 shows the performance of the overall production pipeline (decision rule + Llama 3) on the 100-case test set (target variable prevalence 0.59), highlighting cases where there was independent agreement among reviewers, without the need for group adjudication. The model performed better on cases where experts independently agreed, suggesting that these cases might be easier to classify.

**TABLE 3.** Performance of the Production Pipeline (decision rule + Llama 3) on the Test Set

Metrics^a^	No.	Accuracy	F1 Score	Precision	Recall
Overall	100	0.930	0.941	0.933	0.949
Agreed cases	85	0.965	0.972	1.000	0.946

a The prevalence of the target variable among all cases in the 100-case testing set was 0.59. The prevalence of the target variable among the 85 agreed-upon cases was 0.66.

We also assessed the performance of the five SME reviewers by comparing their answers with the consensus final answers. The mean accuracy across all reviewers was 0.96, with individual accuracies of 0.925, 0.975, 0.963, 0.988, and 0.95.

Our automated classification pipeline (decision rule + Llama 3) achieved an accuracy of 0.930 on the entire test set, surpassing the lowest-performing reviewer's accuracy of 0.925. A chi-square test comparing the reviewers' mean accuracy and our pipeline's performance yielded a *P* value of .188, indicating no significant difference between automated classification and the average reviewer's accuracy (early in this project, we also attempted to apply a rule-based NLP approach, but lacked sufficient labeled data to make such an approach feasible; in initial tests, that approach achieved an accuracy of 0.78 and an F1 score of 0.82).

### Error Analysis

**FIG 3.**
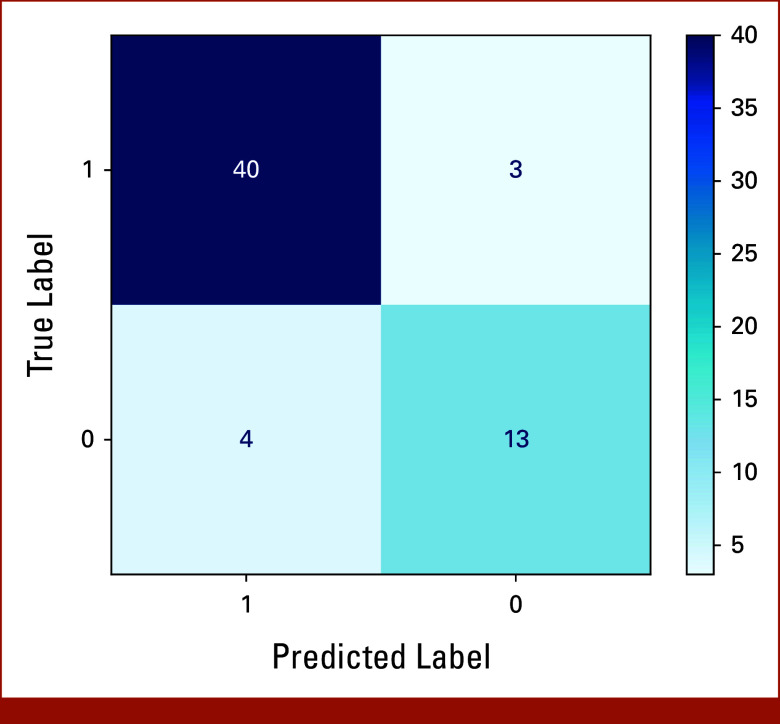
Confusion matrix.

The testing set revealed no errors in classifying patients without any snippets or those with an excessive number of snippets. However, the model misclassified seven cases, four of which involved disagreements among reviewers. Figure 3 presents the confusion matrix for the model-processed cases.

On reviewing model misclassification errors, we observed that these cases were inherently complex and that even domain experts found accurate classification challenging; reviewers disagreed on four of seven misclassified cases. For example, one note stated, “Select all that apply: (1) Genetic testing Yes. (2) Genetic counseling/patient education Yes.” The model classified this as “Yes,” as did one reviewer initially, although reviewers later agreed on classifying it as “Not sure.”

Another note stated, “Daughter really wanted genetic testing done on her mom but profile doesn't really fit given age and what we would do with info. Recommendations Recurrent breast cancer chief complaint: genetic consultation and testing.” The model categorized this as “Not sure,” reasoning that the patient was considered for germline genetic testing but did not meet testing criteria. However, the reviewer classified it as “Yes.” Similar discrepancies were noted in two other cases.

Among the remaining three errors, the model misclassified one as “Not applicable” because the patient had received all breast care in the community. In another case, the model correctly identified that a genetic consult was requested but later canceled because of not meeting criteria, yet it misclassified it “Yes” instead of “No”/“Not sure.” In the final case, the note reported, “Patient states ‘a couple of year ago I did a genetic testing and I was told I was never going to get cancer.’” The model found no evidence of testing and concluded that care was provided in the community, classifying the note as “Not applicable” instead of “Yes.” These errors highlight the inherent difficulty in capturing edge cases through clinical documentation.

## DISCUSSION

Despite the explosion of artificial intelligence (AI) applications in everyday life, attempts to apply AI in health care have so far yielded mixed results.^18,31,32^ Several barriers impede the widespread adoption of AI in health care, including the challenges of integrating AI into existing workflows, the need for high-performance hardware within approved health care data boundaries, and, crucially, the availability of labeled data at scale. Acquiring substantial labeled health care data is often costly, challenging, or, at times, infeasible. For example, in our scenario, labeling just 200 cases required significant effort from already busy clinicians. LLMs like Llama 3 offer a promising alternative to traditional deep learning approaches by performing well even with limited labeled data.

LLMs can also address critical clinical needs, as we have demonstrated. Knowing whether a patient with breast cancer has been provided with germline genetic testing is crucial, given that a significant proportion of breast cancer cases arise from actionable PGVs.^33,34^ However, because of the way this information is documented (ie, in natural language, with results being reported in multiple systems), it is largely inaccessible both to individual clinicians seeking to manage their patients' care and to health care systems seeking to understand quality-of-care delivery. We have shown that LLMs can provide this information accurately and efficiently, without engaging in extensive chart review or trying to change the way this information is documented, which would involve modifying both the medical record system and clinicians' documentation process. LLMs' ability to extract crucial clinical information from unstructured data will likely have far-reaching applications across clinical practice, given that approximately 80% of medical data are unstructured.^35^ It will also enhance researchers' ability to use real-world data, obviating the need to rely on surrogate measures that are easily accessible as structured data but imperfectly represent clinical outcomes.^36^

Our findings underscore the vital role of open-source models in health care, which enable application of LLM technology without needing to share sensitive information with external parties. Although Llama 3 is not updated as frequently as API-based models like GPT-4, its performance closely matches that of human experts.

Technological advances have led to improved LLM performance with smaller model sizes. Here, Llama 3 demonstrated substantial improvements over Llama 2 models, with Llama 3 8B even outperforming Llama 2 70B, defying the common expectation that larger models perform better. This suggests that organizations can select appropriate models based on their specific equipment, budget, and operational needs. We anticipate that future LLMs will provide improved accuracy at reduced costs, further enhancing information extraction tasks in health care compared with human experts.

As LLMs evolve, their ability to handle longer text bodies will make their implementation in health care more practical. Until then, our work demonstrates the benefit of integrating clinical expertise into the design of an LLM pipeline. In this project, we used SMEs' contributions, along with Text Analytics for Health, to minimize text length, reducing processing time and costs for the Llama 2 and Llama 3 models. In the future, we expect LLMs to process longer documents more quickly and affordably.

A limitation of this work was the relatively small amount of labeled data available for validation, which resulted in wider CIs for performance metrics.

In conclusion, our implementation of a state-of-the-art, open-source LLM has addressed a critical knowledge gap within the health care system, enabling a deeper understanding of care quality, while maintaining data protection and minimizing the need for extensive labeled data. Our results highlight significant improvements in performance between the latest Llama 3 model and its predecessor. Looking ahead, we anticipate that LLMs will play a pivotal role in advancing the concept of a learning health system, offering health systems the ability to assess and enhance the quality and efficiency of the care they provide.

## APPENDIX: PROMPT ENGINEERING DETAILS

Following the prompt engineering approach described in the study by Huang et al,^37^ we conducted many experiments to refine our prompt in the prompt engineering process, following the steps outlined below.

### Formatting the Prompts

We first explored different prompt formats. Prompts that required the LLM to provide reasons tended to perform better than prompts that only requested a yes or no answer. Trivial wording changes seemed to bear a significant impact on the performance. We decided to use a prompt with a template to define the desired answer format and provide the reasoning behind the model's decision.

### Clarifying Instructions

We added the instructions our SMEs used for labeling and used four labels in the prompt, yielding an accuracy of 0.88 and an F1 score of 0.92. After reviewing the errors, we added three notes to the prompt; the accuracy improved to 0.93, and the F1 score to 0.95. We further explored combining the “no,” “not applicable,” and “not sure” responses.

### Adding Examples

We added three typical examples in the prompt—two positive cases and one negative case. The accuracy and F1 score dropped to 0.84 and 0.88, respectively.

We decided to return to the optimal prompt for the development set and applied it in the test set.

### Prompt Testing

Below, we demonstrate a few typical prompts and their performance in the development set.

Prompt1

"""

Detection in Text: Look for mentions of germline genetic testing.

Please provide a response in the following format:

Detected Term: [Term]
Relevance to germline genetic testing: [Brief explanation]

If no germline genetic related terms or discussions are found, report: 'No germline genetic testing related content detected."""

Prompt 2

"""

Was this patient offered germline genetic testing for breast cancer?

Please answer: yes or no. Give no extra explanations.

"""

Prompt3

"""

Did this patient receive germline genetic testing for breast cancer?

Please answer: yes or no. Give no extra explanations.

"""

Prompt4

"""

Was this patient tested for germline mutations associated with breast cancer?

Please answer: yes or no. Give no extra explanations.

"""

Prompt5

"""

Please provide your answer in the following format:

Germline genetic testing for breast cancer [was/was not] performed.

"""

Prompt6

"""

Was this patient offered germline genetic testing for breast cancer?

Please provide your answer in the following format:

detection:[yes/no]
reason:[brief reason summary]

"""

### Final Prompt

'role': 'system', 'content': 'You are a medical expert please answer the following question consistently, concisely, scientifically, professionally, focusing on as short as possible answers while following the format provided.'

'role': 'user', 'content': '

Please classify the clinical notes provided into the following categories:

Yes. Had germline genetic testing, recommendation of germline testing, or consult to GMS/CCGS/community care for genetic counseling

AND patient seen by at least one VA provider (med onc, rad onc, surgery), even if patient declines, or the test was not done.

No. Seen by at least one VA provider (med onc, rad onc, surgery) and none of the items in Yes found.
Not Applicable. All of breast cancer care done in the community as shown by patient not seen by at least one VA provider (med onc, rad

onc‹ surgery) regardless of presence of the items in Yes.

Not Sure. Patient see by VA provider and considered for germline genetic testing but testing not done because did not meet criteria for
testing.

Note:

Invitae test is a germline genetic test.
Onco-DX or ONCOTYPE DX test are somatic genetic tests, they are not germline genetic tests.
Answer yes if a consult for genetic medicine is placed or considered.

Which category does the following note belongs to? Please provide your answer in the following format:

category:[yes/no/not applicable/not sure]
reason:[brief reason summary]

**TABLE A1.** Prompt engineering results

Prompt	Accuracy	F1 Score	Precision	Recall
Prompt1	0.86	0.89	0.91	0.87
Prompt2	0.84	0.87	0.97	0.79
Prompt3	0.53	0.46	1	0.3
Prompt4	0.6	0.58	1	0.4
Prompt5	0.57	0.53	1	0.36
Prompt6	0.83	0.86	0.93	0.81
